# Determinants of cord blood adipokines and association with neonatal abdominal adipose tissue distribution

**DOI:** 10.1038/s41366-021-00975-3

**Published:** 2021-12-04

**Authors:** Karen Tan, Mya Thway Tint, Navin Michael, Fabian Yap, Yap Seng Chong, Kok Hian Tan, Keith M. Godfrey, Anis Larbi, Yung Seng Lee, Shiao-Yng Chan, Marielle V. Fortier, Johan G. Eriksson, Neerja Karnani

**Affiliations:** 1 Singapore Institute for Clinical Sciences (SICS), Agency for Science, Technology and Research (A*STAR) Singapore, Singapore, Singapore; 2 Human Potential Translational Research Programme, Yong Loo Lin School of Medicine, National University of Singapore, Singapore, Singapore; 3 Duke-National University of Singapore (NUS) Medical School, Singapore, Singapore; 4 Department of Pediatric Endocrinology, KK Women’s and Children’s Hospital, Singapore, Singapore; 5 National Technological University (NTU)-Lee Kong Chian School of Medicine, Singapore, Singapore; 6 Department of Maternal Fetal Medicine, KK Women’s and Children’s Hospital, Singapore, Singapore; 7 MRC Lifecourse Epidemiology Unit, University of Southampton, Southampton, United Kingdom; 8 NIHR Southampton Biomedical Research Centre, University of Southampton & University Hospital, Southampton, United Kingdom; 9 Singapore Immunology Network (SIgN), Agency for Science, Technology and Research (A*STAR), Singapore, Singapore; 10 Department of Paediatrics, Yong Loo Lin School of Medicine, National University of Singapore, Singapore, Singapore; 11 Khoo Teck Puat – National University Children’s Medical Institute, National University Health System, Singapore, Singapore; 12 Department of Obstetrics and Gynaecology, National University Hospital, Singapore, Singapore; 13 Department of Diagnostic and Interventional Imaging, KK Women’s and Children’s Hospital, Singapore, Singapore; 14 Folkhälsan Research Center, Helsinki, Finland; 15 Department of General Practice and Primary Health Care, University of Helsinki, Helsinki, Finland; 16 Department of Biochemistry, Yong Loo Lin School of Medicine, National University of Singapore, Singapore, Singapore; 17 Bioinformatics Institute (BII), Agency for Science, Technology and Research (A*STAR), Singapore, Singapore

**Keywords:** Obesity, Epidemiology

## Abstract

**Background:**

Cord blood leptin and adiponectin are adipokines known to be associated with birth weight and overall infant adiposity. However, few studies have investigated their associations with abdominal adiposity in neonates. We examined maternal factors associated with cord blood leptin and adiponectin, and the association of these adipokines with neonatal adiposity and abdominal fat distribution measured by magnetic resonance imaging (MRI) in an Asian mother–offspring cohort.

**Methods:**

Growing Up in Singapore Towards healthy Outcomes (GUSTO), is a prospective mother–offspring birth cohort study in Singapore. Cord blood plasma leptin and adiponectin concentrations were measured using Luminex and Enzyme-Linked Immunosorbent Assay respectively in 816 infants. A total of 271 neonates underwent MRI within the first 2-weeks after delivery. Abdominal superficial (sSAT), deep subcutaneous (dSAT), and intra-abdominal (IAT) adipose tissue compartment volumes were quantified from MRI images. Multivariable regression analyses were performed.

**Results:**

Indian or Malay ethnicity, female sex, and gestational age were positively associated with cord blood leptin and adiponectin concentrations. Maternal gestational diabetes (GDM) positively associated with cord blood leptin concentrations but inversely associated with cord blood adiponectin concentrations. Maternal pre-pregnancy body mass index (BMI) showed a positive relationship with cord blood leptin but not with adiponectin concentrations. Each SD increase in cord blood leptin was associated with higher neonatal sSAT, dSAT and IAT; differences in SD (95% CI): 0.258 (0.142, 0.374), 0.386 (0.254, 0.517) and 0.250 (0.118, 0.383), respectively. Similarly, each SD increase in cord blood adiponectin was associated with higher neonatal sSAT and dSAT; differences in SD (95% CI): 0.185 (0.096, 0.274) and 0.173 (0.067, 0.278), respectively. The association between cord blood adiponectin and neonatal adiposity was observed in neonates of obese mothers only.

**Conclusions:**

Cord blood leptin and adiponectin concentrations were associated with ethnicity, maternal BMI and GDM, sex and gestational age. Both adipokines showed positive association with neonatal abdominal adiposity.

## Introduction

Adipokines are hormones secreted by adipocytes and suggested to play important roles in energy homeostasis and cell metabolism [1]. Leptin, a peptide hormone produced by adipocytes in proportion to their triglyceride content, connects fat stores with the central control of energy balance [2]. Although leptin deficiency results in increased food intake and decreased energy expenditure [3], most obese individuals have elevated leptin concentrations which is thought to be a leptin resistant state [2]. Adult leptin concentrations are higher in Indians and Malays compared to Chinese [4]. Many studies have shown positive associations of cord blood leptin and birth weight [5–10]. Maternal characteristics, such as obesity [7], smoking [10] and glucose concentrations [11], have been associated with increased cord blood leptin concentrations. Fetal leptin has been proposed to influence the programming of hypothalamic neuronal networks to influence adiposity in the long term [12]. Several studies have examined the association of cord blood leptin with adiposity in later childhood [8, 13–16] and a report has suggested that this association changes as the child grows with age [14].

Adiponectin, one of the adipokines produced by adipocytes, mediates communication of the adipose tissue with other metabolic tissues such as the liver and skeletal muscle [17]. Adiponectin suppresses hepatic gluconeogenesis and promotes insulin sensitization in adults [17, 18]. In adults, lower adiponectin concentrations are associated with adverse metabolic parameters such as greater adiposity, insulin resistance and gestational diabetes mellitus (GDM) [18–20], and Indian ethnicity compared to Malay and Chinese [21]. On the other hand, prenatal exposures such as maternal obesity [22] and GDM status [23] have been associated with higher cord blood adiponectin concentrations. Cord blood adiponectin concentration was shown to be positively associated with cord blood leptin concentration, fetal growth and birthweight [9, 24–26], and fetal adiponectin concentration is higher than adult adiponectin concentration [27, 28]. Maternal adiponectin concentrations have shown to be inversely associated with offspring’s birth weight and adiposity measures [20, 29, 30]. During pregnancy, adiponectin levels decrease as the mother develops an insulin-resistant state to support reduced glucose uptake and increased lipolysis, shifting nutrients such as glucose and lipids to the fetus [29, 31]. A high concentration of adiponectin in the fetus may enhance the growth‐promoting effect of insulin through its insulin‐sensitizing action [9]. Studies in mice have shown that fetal adiponectin enhances fetal fat deposition and plays a role in maternal obesity-induced increased birth weight [32].

Increased abdominal adiposity (AA) is a known independent risk factor for adverse cardio-metabolic outcomes in adults [33]. Increased visceral fat deposition in older children and adolescents has also been associated with insulin resistance, and an increased risk of cardiovascular disease and diabetes [34, 35]. South Asians are characterized by greater abdominal obesity and higher insulin resistance as compared to Caucasians of similar body mass index (BMI) [36]. We have observed differential distribution of abdominal adipose tissue compartments (AAT) in neonates among Asian ethnic groups in Singapore [37], with higher abdominal subcutaneous adipose tissue among Indian neonates despite lower birth weight. This finding is in line with the notion that Indian newborns are born light but with higher body fat [38]. With the increasing prevalence of childhood obesity [39] and the prevalence of metabolic syndrome among children and adolescents [40], it is important to better understand the early life factors influencing AA.

Both cord blood leptin and adiponectin have been reported to be positively associated with birth weight and overall adiposity at birth measured mostly by simple anthropometry such as BMI and skinfold thickness [6, 7, 9–11, 14, 24, 25, 28]. However, although leptin is known to be correlated with AA [13–16], few of these studies have included more accurate measurements of AA compartment volumes by MRI in early infancy. This study aimed to determine [1] maternal and fetal determinants of cord blood leptin and adiponectin concentrations in Asian neonates, and [2] the association between cord blood leptin and adiponectin concentrations and AA, as measured by MRI, in early infancy in a multi-ethnic Asian prospective cohort.

## Methods

### Study design and population

The study was based on mother–offspring pairs from the Growing Up in Singapore Towards healthy Outcomes (GUSTO) study, a prospective cohort study in Singapore [41]. Pregnant women aged 18 years and above were recruited between June 2009 and September 2010 during the first trimester of pregnancy (< 14 weeks’ gestation based on dating ultrasound scan) from two public maternity units in Singapore; KK Women’s and Children’s Hospital (KKH) and National University Hospital (NUH). This study was approved by the Institutional Review Board of the Singapore National Healthcare Group and the Central Institutional Review Board of Singhealth. Parents of the neonates provided voluntary written consent for the study. This study was registered at clinicaltrials.gov as NCT01174875.

### Maternal characteristics

Demographic data, lifestyle, obstetric and medical history were collected at multiple study visits using interviewer administered questionnaires and from hospital records. Self-reported pre-pregnancy weights of mothers were recorded. Pre-pregnancy body mass index (ppBMI) was calculated from the self-reported pre-pregnancy weight and measured height at booking. ppBMI categories were defined using WHO recommended BMI cutoff points for Asians [42]. Pregnant women underwent a 2 h 75 g oral glucose tolerance test (OGTT) at 26–28 weeks gestation. Glucose concentrations were measured using hexokinase method (Advia 2400 Chemistry system (Siemens Medical Solutions Diagnostics) and Beckman LX20 Pro analyzer (Beckman Coulter)). GDM was diagnosed using 1999 World Health Organization (WHO) criteria: ≥7.0 mmol/L for fasting plasma glucose or ≥7.8 mmol/l for 2 h post-prandial glucose [43]. Gestational weight gain (GWG) groups were defined by 1990 Institute of Medicine guidelines (IOM) for rates of weight gain in the second and third trimester per week [44].

### Infant measurements

Information on birth weight, birth length, and head circumference were obtained from medical records. Triceps and subscapular skinfolds were measured on the right side in triplicates to the nearest 0.2 mm using Holtain skinfold calipers (Holtain Ltd, Crymych, UK). The validated GUSTO equation for estimating fat mass (Fat Mass_GUSTO_ = −0.022 + (0.307 × weight) − (0.077 × gender) + (0.028 × subscapular skinfolds) − (0.019 × gestational age), where gender = 1 for male, 0 for female) was used to calculate fat mass [45]. The PEA POD^®^ Infant Body Composition System Version 3.1.0 (Cosmed, Italy) was used to measure body composition, i.e., fat mass and fat free mass [46, 47]. After excluding neonates whose parents did not consent for PEA POD^®^ measurement, and those that had <2.5 kg birthweight, <5% percent body fat in PEA POD^®^ assessment [45], and no cord blood adipokines measured, a total of 259 neonates remained for subsequent analysis on PEA POD^®^ – adipokine association. Clothing was removed from the infant and the infant was placed on the scale for body mass measurement and inside the chamber for body volume measurement. Percent body fat was computed by the PEA POD system. Age and sex-specific densities of free fat mass based on multi-compartment studies are used by the PEA POD^®^ [45].

### Cord blood adipokine concentrations

Umbilical cord blood leptin was measured in EDTA plasma from venous cord blood using the Procarta-5-plex-DropArray Luminex assay. The coefficient of variation (CV) of a pooled plasma control was 17.3%. Umbilical cord blood total adiponectin was measured in EDTA plasma from venous cord blood using the Adiponectin Human in vitro ELISA (Enzyme-Linked Immunosorbent Assay) kit (Abcam). The CV of a pooled plasma control was 13.3%. Adjustment for plate effect was performed using median centering where the median of each plate was shifted to the global median for all plates.

### Quantification of abdominal adipose tissue compartments

333 healthy neonates born ≥34 weeks gestation with birth weight (BW) ≥ 2000g had abdominal adipose tissue compartment (AAT) volumes data available from MRI scans performed within two weeks post-birth. A detailed participant selection flow chart can be accessed from Tint MT et al. [37]. Of these 333 neonates, cord blood adipokines data was available for 271 neonates. Briefly, non-sedated neonates were placed in an immobilization bag within an adult head coil. The abdomen was scanned from the diaphragm to the symphysis pubis. T1-weighted water-suppressed (WS) and non-WS axial fast-spin echo sequences were acquired by GE Signa HDxt 1.5 tesla magnetic resonance scanner (GE Healthcare). Pulse and oxygen saturation amounts of the neonate were monitored in the presence of a neonatologist. WS images were processed to yield quantitative values of AAT volumes. Non-WS images were used to assist in the localization of anatomical structures if necessary. The AAT was categorized into superficial (sSAT), deep (dSAT) subcutaneous and internal (IAT) adipose tissue. MRI images were processed by an in-house semi-automated quantitative analysis algorithm using MATLAB 7.13 software (The MathWorks Inc., Natick, Massachusetts, USA). All MRI images were analyzed by a physician and an experienced magnetic resonance analyst.

### Statistical analysis

The characteristics of mothers and offspring were compared among the whole cohort vs. MRI and PEAPOD^®^ subsets using independent sample *T*-tests for continuous variables and chi square tests for categorical variables. Cord blood leptin and adiponectin concentrations were not normally distributed thus Mann–Whitney U tests were used for group comparison.

Multivariable regression analyses were performed to determine maternal and neonatal factors associated with cord blood adipokines. Outcome variables i.e., adipokine concentrations were transformed into standardized scores so that the strength of associations were comparable in regression model. Models were mutually adjusted for ethnicity, maternal education, tobacco exposure, parity, maternal age, maternal pre-pregnancy BMI, GWG categories, GDM status, gestational age at delivery and child’s sex.

Multivariable regression analyses were performed with cord blood adipokines (leptin and adiponectin) as the main exposures, birth weight, skinfold thickness measurement as total adiposity measure and AAT compartment volumes as outcomes of interest. All exposures and outcome variables were transformed into standardized scores so that observed strength of associations were comparable. Covariates were controlled for based on prior knowledge from the literature about factors that might confound the associations between maternal and cord adipokines and neonatal adiposity. Models were adjusted for ethnicity, pre-pregnancy BMI, GDM status, gestational age at delivery, and child’s sex and birth length. When studying neonatal adiposity by MRI, models were additionally adjusted for age on MRI day. *P* values were corrected using Benjamini–Hockberg method with false discovery rate of 0.05(25) [48]. All statistical analyses used SPSS Statistics for Windows, Version 23.0. (IBM Corp., Armonk, NY).

## Results

### Distribution of cord blood adipokines

A flow chart of this study is shown in Supplementary Fig. 1. The characteristics of the study participants (whole cohort vs. MRI and PEA POD subsets) are shown in Supplementary Table 1. The mothers of the neonates who underwent MRI and PEA POD measurements were slightly younger, with lower 2 h post-OGTT glucose concentrations compared to mothers of the neonates who did not undergo MRI and PEA POD measurements, and with a greater proportion of Malay participants, more mothers who smoked, and a lower proportion of mothers with higher education. Cord blood leptin concentrations ranged from 0.3 to 20.4 ng/mL and cord adiponectin concentrations from 0.3 to 28.4 µg/mL. There was a positive correlation between cord leptin and adiponectin (*r* = 0.103, *p* = 0.003).

### Factors associated with cord blood adipokine concentrations

Factors associated with cord blood leptin and adiponectin are shown in Table 1. Female sex and longer gestation were associated with higher cord blood leptin and adiponectin concentrations. Indian and Malay neonates had higher cord blood leptin concentrations compared to Chinese neonates, while Malay neonates had higher cord blood adiponectin concentrations compared to Chinese neonates. Infants of mothers with GDM had higher cord blood leptin concentrations but lower cord blood adiponectin concentrations compared to those offspring of mothers without GDM. In addition, mothers with ppBMI in the overweight category had neonates with higher cord blood leptin concentrations.

**Table 1:** Factors associated with cord blood leptin and adiponectin.

	*N* = 565	Cord blood Leptin	*P*	Cord blood Adiponectin	*P*
		β (95% CI)		β (95% CI)	
Ethnicity					
Chinese	285	Reference		Reference	
Malay	170	0.229 (0.027, 0.431)	0.026	0.268 (0.050, 0.486)	0.016
Indian	110	0.628 (0.409, 0.847)	<0.001	0.213 (−0.023, 0.449)	0.076
Gestational diabetes mellitus (GDM)					
No	470	Reference		Reference	
Yes	95	0.418 (0.198, 0.637)	<0.001	−0.243 (−0.481, −0.005)	0.045
Mother pre-pregnancy BMI category					
BMI < 18.5 kg/m^2^	61	−0.048 (−0.315, 0.219)	0.724	−0.002 (−0.292, 0.287)	0.989
BMI 18.5–22.9 kg/m^2^	281	Reference		Reference	
BMI 23.0–24.9 kg/m^2^	74	0.256 (0.009, 0.504)	0.042	−0.074 (−0.340, 0.192)	0.586
BMI ≥ 25.0 kg/m^2^	149	0.143 (−0.067, 0.352)	0.182	0.196 (−0.029, 0.422)	0.088
Gestational age at delivery (weeks)	565	0.180 (0.121, 0.238)	<0.001	0.117 (0.054, 0.180)	<0.001
Sex					
Male	291	Reference		Reference	
Female	274	0.379 (0.221, 0.536)	<0.001	0.254 (0.084, 0.423)	0.003
Education					
Primary	26	0.079 (−0.336, 0.494)	0.708	−0.315 (−0.763, 0.132)	0.400
Secondary	369	0.122 (−0.069, 0.312)	0.210	0.061 (−0.144, 0.267)	0.559
Tertiary	170	Reference		Reference	
Tobacco Exposure					
No exposure	285	Reference		Reference	
Exposed with cotinine level <level of detection	173	0.213 (−0.259, 0.685)	0.376	0.036 (−0.472, 0.544)	0.890
Exposed with cotinine level <14 ng/ml	89	0.045 (−0.203, 0.293)	0.720	0.092 (−0.175, 0.360)	0.497
Exposed with cotinine level ≥14 ng/ml	18	0.048 (−0.145, 0.241)	0.625	0.060 (−0.148, 0.269)	0.570
Parity					
Multiparity	327	Reference		Reference	
Primiparity	238	0.031 (−0.145, 0.207)	0.728	−0.135 (−0.324, 0.054)	0.161
Gestational weight gain (GWG) categories based on 1999 IOM gudelines					
Optimal	195	Reference		Reference	
Inadequate	68	−0.146 (−0.412, 0.121)	0.284	0.169 (−0.118, 0.456)	0.247
Excessive	302	0.074 (−0.111, 0.259)	0.433	0.059 (−0.140, 0.259)	0.560
Maternal Age (Years)	565	0.011 (−0.006, 0.028)	0.219	0.002 (−0.017, 0.020)	0.875

Standardized scores of cord blood leptin and adiponectin as outcomes. Coefficients (β) are change in independent variables with 95% confidence intervals (95% CI) per standardized score value change in cord blood leptin or adiponectin. *P* values were determined with the use of multivariable regression models. Models are mutually adjusted for ethnicity, maternal education, tobacco exposure, parity, maternal age, maternal pre-pregnancy BMI categories, gestational weight gain (GWG) categories based on 1999 the Institute of Medicine (IOM) guideline, gestational diabetes mellitus (GDM) status, gestational age at delivery and child’s sex.

### Associations between cord blood adipokines and neonatal adiposity

Table 2 shows the associations between cord adipokines and neonatal adiposity. Higher cord blood leptin and adiponectin concentrations were associated with higher birth weight, skinfold thicknesses, fat mass, and higher AAT measured by MRI. Each SD increase in cord blood leptin was associated with a 0.212 (0.259, 0.266) SD increase in birth weight and a 0.378 (0.237, 0.519) SD increase in fat mass in the neonates. Each SD increase in cord blood leptin was associated with 0.258 (0.142, 0.374), 0.386 (0.254, 0.517) and 0.250 (0.118, 0.383) SD increases in sSAT, dSAT and IAT, respectively.

**Table 2:** Association between cord blood leptin and adiponectin and neonatal adiposity.

	Birth weight (*N* = 650)	Triceps skinfold (*N* = 626)	Subscapular skinfold (*N* = 625)	Fat Mass (PEAPOD) (*N* = 197)	Fat Mass (predicted) (*N* = 625)	sSAT (*N* = 213)	dSAT (*N* = 213)	IAT (*N* = 213)
	β (95% CI)	β (95% CI)	β (95% CI)	β (95% CI)	β (95% CI)	β (95% CI)	β (95% CI)	β (95% CI)
Cord blood Leptin	0.212 (0.259, 0.266)	0.267 (0.186, 0.349)	0.319 (0.238, 0.400)	0.378 (0.237, 0.519)	0.281(0.218, 0.343)	0.258 (0.142, 0.374)	0.386 (0.254, 0.517)	0.250 (0.118, 0.383)
	***P*** < **0.001**^**a**^	***P*** < **0.001**^**a**^	***P*** < **0.001**^**a**^	***P*** < **0.001**^**a**^	***P*** < **0.001**^**a**^	***P*** < **0.001**^**b**^	***P*** < **0.001**^**b**^	***P*** = **0.001**^**b**^
Cord blood Adiponectin	0.110 (0.059, 0.162)	0.158 (0.080, 0.235)	0.179 (0.102, 0.256)	0.136 (0.014, 0.257)	0.154 (0.094, 0.215)	0.185 (0.096, 0.274)	0.173 (0.067, 0.278)	0.092 (−0.011, 0.195)
	***P*** < **0.001**^**a**^	***P*** < **0.001**^**a**^	***P*** < **0.001**^**a**^	***P*** = **0.033**^**a**^	***P*** < **0.001**^**a**^	***P*** < **0.001**^**b**^	***P*** = **0.002**^**b**^	*P* = 0.081^b^

Standardized scores of cord blood leptin and adiponectin as exposures. Standardized scores of birth weight, triceps skinfold, subscapular skinfold, fat mass,

sSAT, dSAT, and IAT as outcomes.

Coefficients (β) with 95% confidence intervals (95% CI) are change in independent variables per standardized score value change in cord blood leptin or adiponectin. *P* values were determined with the use of multivariable regression models and corrected for multiple analyses using Benjamini–Hockberg method. Significant *p*-values < 0.05 are indicated in bold.

*sSAT* Abdominal superficial subcutaneous adipose tissue, *dSAT* Abdominal deep subcutaneous adipose tissue, *IAT* Abdominal internal adipose tissue.

^a^Models are adjusted for ethnicity, pre-pregnancy BMI, gestational diabetes mellitus (GDM) status, gestational age at delivery, and child’s sex and birth length.

^b^Models are adjusted for ethnicity, pre-pregnancy BMI, GDM status, gestational age at delivery, and child’s sex, birth length and age on MRI day.

Similarly, each SD increase in cord blood adiponectin was associated with a 0.110 (0.059, 0.162) SD increase in birth weight and a 0.136 (0.014, 0.257) SD increase in fat mass by PEAPOD in neonates. Each SD increase in cord blood adiponectin was associated with 0.185 (0.096, 0.274), 0.173 (0.067, 0.278) and 0.092 (−0.011, 0.195) SD increases in neonatal sSAT, dSAT and IAT, respectively.

Table 3 shows the associations between cord adipokines and neonatal adiposity stratified by maternal BMI categories; underweight, normal weight, overweight and obese. Neonates of mothers of all BMI categories showed a significant positive association between cord leptin concentrations and birth weight as well as measures of neonatal adiposity. In contrast, only neonates of obese mothers with BMI ≥ 25 kg/m^2^ showed statistically significant positive association between cord adiponectin concentrations and birthweight as well as measures of neonatal adiposity. The interaction term was significant (*p* < 0.05) for the interaction between obese maternal BMI category and the cord adiponectin concentrations in the association with predicted fat mass and sSAT (Table 3).

**Table 3:** Association between cord blood leptin and adiponectin and neonatal adiposity stratified by pre-pregnancy BMI.

Underweight	Birth weight (*N* = 79)	Triceps skinfold (*N* = 77)	Subscapular skinfold (*N* = 76)	Fat Mass (PEAPOD) (*N* = 25)	Fat Mass (predicted) (*N* = 76)	sSAT (*N* = 29)	dSAT (*N* = 29)	IAT (*N* = 29)
BMI < 18.5 kg/m^2^	β (95% CI)	β (95% CI)	β (95% CI)	β (95% CI)	β (95% CI)	β (95% CI)	β (95% CI)	β (95% CI)
Cord blood Leptin	0.283 (0.111, 0.455)	0.500 (0.253, 0.747)	0.462 (0.178, 0.745)	0.711 (0.163, 1.259)	0.429 (0.209, 0.650)	0.280 (−0.028, 0.588)	0.560 (0.093, 1.027)	0.396 (0.049, 0.744)
	***P*** = **0.004**^**a**^	***P*** < **0.001**^**a**^	***P*** = **0.004**^**a**^	***P*** = **0.023**^**a**^	***P*** < **0.001**^**a**^	*P* = 0.072^b^	***P*** = **0.028**^**b**^	***P*** = **0.031**^**b**^
*p* value for interaction	0.271	0.517	0.807	0.985	0.256	0.914	0.758	0.108
Cord blood Adiponectin	0.153 (−0.024, 0.331)	0.151 (−0.110, 0.412)	0.375 (0.133, 0.617)	−0.153 (−0.793, 0.486)	0.235 (0.037, 0.432)	0.081 (−0.151, 0.313)	0.018 (−0.361, 0.397)	−0.105 (−0.387, 0.177)
	*P* = 0.239^a^	*P* = 0.503^a^	***P*** = **0.023**^**a**^	*P* = 0.708^a^	*P* = 0.083^a^	*P* = 0.636^b^	*P* = 0.921^b^	*P* = 0.636^b^
*p* value for interaction	0.238	0.399	**0.028**	0.067	0.105	0.981	0.73	0.497

*Normal weight*	Birth weight (*N* = 318)	Triceps skinfold (*N* = 306)	Subscapular skinfold (*N* = 306)	Fat Mass (PEAPOD) (*N* = 95)	Fat Mass (predicted) (*N* = 306)	sSAT (*N* = 91)	dSAT (*N* = 91)	IAT (*N* = 91)
BMI 18.5–22.9 kg/m^2^	β (95% CI)	β (95% CI)	β (95% CI)	β (95% CI)	β (95% CI)	β (95% CI)	β (95% CI)	β (95% CI)
Cord blood Leptin	0.186 (0.103, 0.270)	0.336 (0.207, 0.465)	0.391 (0.275, 0.506)	0.467 (0.239, 0.696)	0.272 (0.177, 0.367)	0.325 (0.123, 0.527)	0.375 (0.141, 0.608)	0.139 (−0.084, 0.363)
	***P*** < **0.001**^**a**^	***P*** < **0.001**^**a**^	***P*** < **0.001**^**a**^	***P*** < **0.001**^**a**^	***P*** < **0.001**^**a**^	***P*** = **0.002**^**b**^	***P*** = **0.002**^**b**^	*P* = 0.218^b^
*p* value for interaction	Reference	Reference	Reference	Reference	Reference	Reference	Reference	Reference
Cord blood Adiponectin	0.055 (−0.019, 0.129)	0.078 (−0.040, 0.196)	0.104 (−0.004, 0.212)	0.274 (0.089, 0.460)	0.080 (−0.007, 0.168)	0.085 (−0.058, 0.227)	0.140 (−0.023, 0.304)	−0.016 (−0.167, 0.135)
	*P* = 0.234^a^	*P* = 0.260^a^	*P* = 0.183^a^	***P*** = **0.034**^**a**^	*P* = 0.183^a^	*P* = 0.276^b^	*P* = 0.183^b^	*P* = 0.835^b^
*p* value for interaction	Reference	Reference	Reference	Reference	Reference	Reference	Reference	Reference

*Overweight*	Birth weight (*N* = 77)	Triceps skinfold (*N* = 73)	Subscapular skinfold (*N* = 73)	Fat Mass (PEAPOD) (*N* = 16)	Fat Mass (predicted) (*N* = 73)	sSAT (*N* = 29)	dSAT (*N* = 29)	IAT (*N* = 29)
BMI 23.0–24.9 kg/m^2^	β (95% CI)	β (95% CI)	β (95% CI)	β (95% CI)	β (95% CI)	β (95% CI)	β (95% CI)	β (95% CI)
Cord blood Leptin	0.183 (0.037, 0.329)	0.267 (0.005, 0.530)	0.231 (−0.084, 0.546)	0.485 (−0.172, 1.142)	0.218 (0.027, 0.409)	0.595 (0.240, 0.950)	0.770 (0.353, 1.188)	0.797 (0.394, 1.199)
	***P*** = **0.030**^**a**^	*P* = 0.061^a^	*P* = 0.148^a^	*P* = 0.142^a^	***P*** = **0.041**^**a**^	***P*** = **0.006**^**b**^	***P*** = **0.004**^**b**^	***P*** = **0.004**^**b**^
*p* value for interaction	0.423	0.941	0.946	0.487	0.61	0.072	**0.037**	**0.001**
Cord blood Adiponectin	0.141 (−0.020, 0.302)	0.207 (−0.074, 0.488)	0.181 (−0.156, 0.519)	0.447 (−0.138, 1.031)	0.179 (−0.031, 0.388)	0.452 (−0.019, 0.523)	0.363 (−0.221, 0.946)	0.498 (−0.050, 1.045)
	*P* = 0.185^a^	*P* = 0.194^a^	*p* = 0.287^a^	*P* = 0.185^a^	*P* = 0.185^a^	*P* = 0.185^b^	*P* = 0.239^b^	*P* = 0.185^b^
*p* value for interaction	0.578	0.620	0.706	0.998	0.799	0.971	0.499	0.545

*Obese*	Birth weight (*N* = 176)	Triceps skinfold (*N* = 170)	Subscapular skinfold (*N* = 170)	Fat Mass (PEAPOD) (*N* = 61)	Fat Mass (predicted) (*N* = 170)	sSAT (*N* = 64)	dSAT (*N* = 64)	IAT (*N* = 64)
BMI ≥ 25.0 kg/m^2^	β (95% CI)	β (95% CI)	β (95% CI)	β (95% CI)	β (95% CI)	β (95% CI)	β (95% CI)	β (95% CI)
Cord blood Leptin	0.210 (0.109, 0.312)	0.128 (−0.013, 0.269)	0.199 (0.063, 0.335)	0.203 (−0.028, 0.435)	0.246 (0.133, 0.359)	0.152 (−0.049, 0.353)	0.288 (0.064, 0.512)	0.099 (−0.145, 0.342)
	***P*** < **0.001**^**a**^	*P* = 0.112^a^	***P*** = **0.012**^**a**^	*P* = 0.112^a^	***P*** < **0.001**^**a**^	*P* = 0.155^b^	***P*** = **0.025**^**b**^	*P* = 0.422^b^
*p* value for interaction	0.839	0.075	0.059	0.082	0.613	0.635	0.900	0.961
Cord blood Adiponectin	0.169 (0.076, 0.262)	0.230 (0.102, 0.358)	0.246 (0.122, 0.371)	0.064 (−0.116, 0.244)	0.233 (0.130, 0.337)	0.278 (0.138, 0.418)	0.230 (0.058, 0.403)	0.214 (0.035, 0.393)
	***P*** < **0.001**^**a**^	***P*** < **0.001**^**a**^	***P*** < **0.001**^**a**^	*P* = 0.480^a^	***P*** < **0.001**^**a**^	***P*** < **0.001**^**b**^	***P*** = **0.013**^**b**^	***P*** = **0.023**^**b**^
*p* value for interaction	0.053	0.056	0.104	0.123	**0.035**	**0.028**	0.412	**0.032**

Standardized scores of cord blood leptin and adiponectin as exposures. Standardized scores of birth weight, triceps skinfold, subscapular skinfold, fat mass, sSAT, dSAT, and IAT as outcomes.

Coefficients (β) with 95% confidence intervals (95% CI) are change in independent variables per standardized score value change in cord blood leptin or adiponectin. *P* values were determined with the use of multivariable regression models and corrected for multiple analyses using Benjamini–Hockberg method. Significant *p*-values < 0.05 are indicated in bold.

*sSAT* Abdominal superficial subcutaneous adipose tissue, *dSAT* Abdominal deep subcutaneous adipose tissue, *IAT* Abdominal internal adipose tissue.

^a^Models are adjusted for ethnicity, gestational diabetes mellitus (GDM) status, gestational age at delivery, and child’s sex and birth length.

^b^Models are adjusted for ethnicity, GDM status, gestational age at delivery, and child’s sex, birth length and age on MRI day.

## Discussion

In this study, we examined the factors associated with cord blood leptin and adiponectin concentrations and studied the association between cord adipokines and neonatal AA. We found that sex, ethnicity, gestational age, maternal adiposity and GDM were associated with cord blood adipokine concentrations and both cord leptin and adiponectin associated positively with neonatal AA.

Maternal GDM was associated with higher cord blood leptin but lower cord blood adiponectin concentrations, similar to previous reports [49–51]. This is consistent with our previous findings that maternal glycaemia is an important determinant of neonatal adiposity [52]. We have observed a positive association between maternal glycemia and neonatal AA across a continuum of glucose levels [52], and postulate that fetal adipokines may play a role in the association between maternal glycaemia and neonatal AA status. Maternal hyperglycemia is linked to fetal hyperinsulinemia and risk of obesity in later life [53]. Our finding that maternal gestational diabetes associates with cord adipokines suggest fetal programming of fetal adipocytes by maternal hyperglycemia. Maternal glycemia is associated with increased maternal leptin, which may contribute to increased cord blood leptin and fetal growth [54]. On the other hand, high maternal glycemia increases maternal insulin resistance, and the reduced insulin signaling may contribute to reduced fetal growth which triggers an increase in fetal adiponectin concentration [31].

We observed ethnic differences in both cord blood leptin and adiponectin concentrations. Cord blood leptin concentrations were higher in Indian neonates compared to Chinese neonates, while cord blood adiponectin concentrations were higher in Malay neonates compared to Chinese neonates. Perhaps this reflects our previous observations in the GUSTO cohort that Indian and Malay neonates had greater metabolically active dSAT volumes compared to Chinese neonates [37]. The ethnic differences in adipokines may in part contribute to observed differences in neonatal adiposity between ethnic groups.

We found that female neonates had higher concentrations of both leptin and adiponectin in cord blood compared to male neonates. This aligns with our finding that female infants have higher sSAT and dSAT volumes than male infants [37]. Most studies have reported higher cord leptin but not cord blood adiponectin concentrations in female infants [53, 55, 56].

The positive association between cord blood leptin concentrations and neonatal adiposity in this study is consistent with previous studies on birth weight and other measures of adiposity [5, 9, 55]. The production of leptin by the placenta in early fetal development suggests that besides being an indicator of fetal adipose tissue, leptin plays an important role in fetal growth [9]. The association between cord blood leptin concentration and neonatal adiposity was stronger than that for cord blood adiponectin concentration in this study, similar to other studies [13, 14, 55]. The strongest associations among all the adiposity outcomes was between dSAT and fat mass and cord blood leptin concentrations. dSAT is most metabolically active and similar to visceral fat, as well as highest in Indians [37], therefore higher cord blood leptin may predispose the neonate to increased metabolic risk in future. Unlike in adults, higher cord blood adiponectin was associated with higher neonatal adiposity. However, our findings are consistent with previous studies [9, 55, 57, 58]. The lack of association between cord adiponectin and IAT is interesting as it is consistent with studies showing an association between adiponectin with subcutaneous fat rather than visceral fat [59, 60].

The positive association between cord blood adiponectin concentrations and neonatal adiposity in this study is also consistent with previous studies [9, 24–28]. The mechanism for the positive association between adiponectin and adiposity in the neonate compared to a negative association in adults is still unknown, and may be due to differences in metabolic function of adipocytes and secretion of adipokines [24]. Cord blood leptin and adiponectin correlated positively with each other, suggesting that fetal leptin may play a role in the secretion of adiponectin in the fetus, which then act in synergy to regulate fetal growth [9]. The observation that cord adiponectin associated with higher birthweight and neonatal adiposity only in obese mothers suggests that the associations may be different between women who become pregnant when obese and those who become pregnant when lean. Women who are obese may have higher insulin resistance corresponding to enhanced allocation of nutrients to the fetus [31]. The increased adiponectin in the cord blood may then enhance the growth‐promoting effect of insulin through its insulin‐sensitizing action, resulting in increased birthweight and adiposity [9]. Cord adipokines are likely associated with multiple pathways that influence fetal growth and the mechanisms by which maternal obesity influences these pathways require further investigation in human studies.

Other studies have described the positive association between cord blood adipokines and adiposity in early childhood [13–16, 24, 25, 55]. However several studies suggested that cord blood adipokines may not predict later childhood obesity [13–15, 25]. Longitudinal studies with follow-up of the GUSTO children are needed to explore the association between cord adipokines and childhood growth and adiposity. High molecular weight adiponectin (HMW adiponectin) is thought to be a better predictor of obesity related metabolic parameters in children [61, 62]. In this study only total adiponectin was measured, however HMW adiponectin has been shown to be a major contributor to total adiponectin in cord blood and cord HMW adiponectin and total adiponectin have been shown to correlate well with each other [25, 63, 64].

Our study is one of the first to use MRI to quantify AAT compartments in early infancy. GUSTO is a prospective study of Asian mothers and offspring pairs, including three Asian ethnic populations (Chinese, Malays, Indians) covering around 50% of the global population. While most previous studies used total adiposity of offspring in association with adipokine concentrations, we used AAT quantified from MRI, an accurate method without radiation to quantify AA, which has been shown to be related to metabolic health. The timing of MRI scans for neonates was within 2-weeks after delivery thus the observation would largely reflect the developmental influences on the offspring before any postnatal environmental exposures.

Our study has some limitations. One is that only a subset of eligible neonates whose parents gave consent for their MRI were included in the study on AA, so care therefore should be taken not to generalize our findings. Future studies are warranted to confirm these findings in larger study populations. Furthermore, as with any other observational studies, we cannot completely exclude bias due to residual confounding although major potential confounders were adjusted for in the study.

In summary, we show that [1] fetal adipokines are associated with maternal factors such as BMI and GDM and there are ethnic and gender differences in cord adipokine levels in Asian neonates, and [2] cord blood leptin and adiponectin positively correlate with measures of neonatal abdominal fat accumulation. Further research is required to determine if these findings have an impact on early childhood abdominal fat accumulation.

## Supplementary information

Supplementary data file
