# Supplementary material for: Determinants of cord blood adipokines and association with neonatal abdominal adipose tissue distribution
Source: Int J Obes (Lond). 2021 Dec 4;46(3):637–45. doi: 10.1038/s41366-021-00975-3 (PMC8873009; doi:10.1038/s41366-021-00975-3)
Supplement: Supplementary file 1 — Supplementary data file [file 41366_2021_975_MOESM1_ESM.docx]

**Supplementary Material**

**Supplementary Figure 1** Flow diagram of the study participants

**
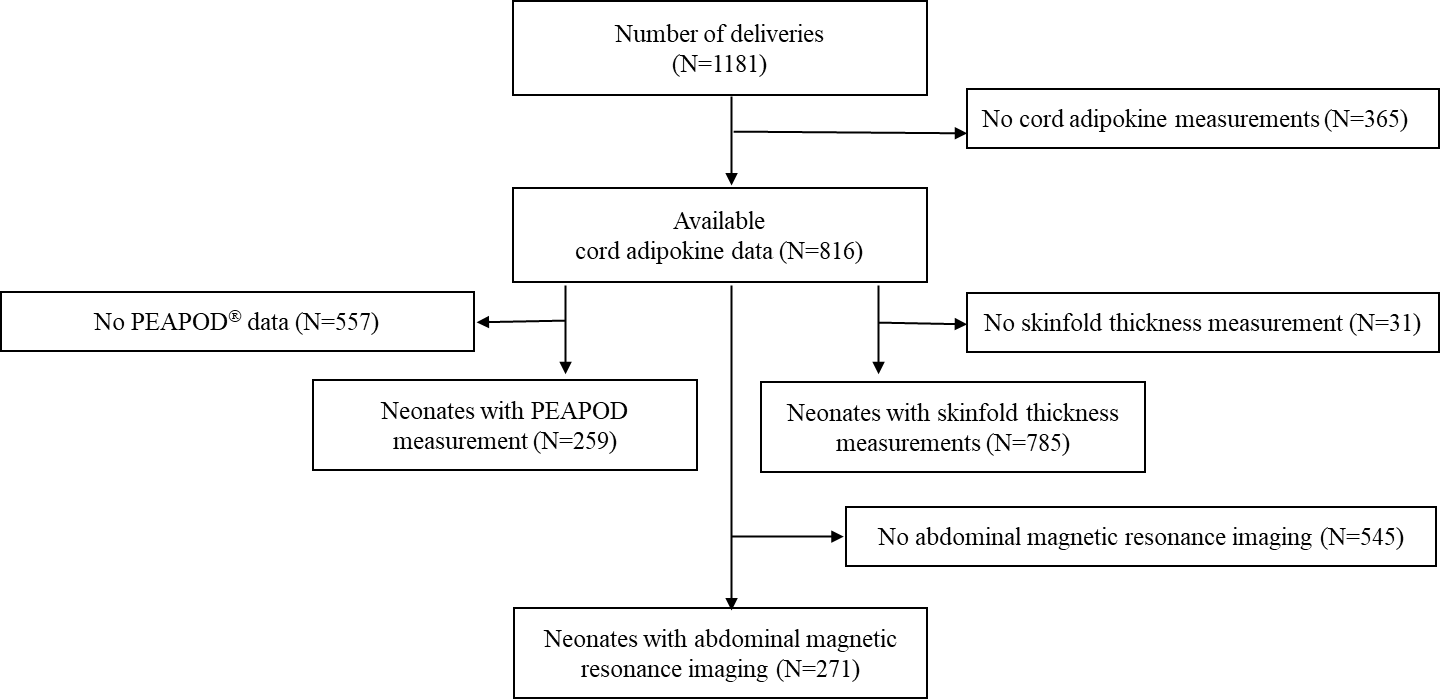
**

**Supplementary Table 1** Maternal and offspring characteristics among study participants

|  | Study participants | Participants with available MRI data |  | Participants with available PEA POD data |  |
| --- | --- | --- | --- | --- | --- |
|  | (N=816) | (N=271) | P | (N=259) | P |
| Maternal characteristics |  |  |  |  |  |
| Ethnicity |  |  | 0.009 |  | 0.004 |
| Chinese | 414 (50.7) | 108 (39.9) |  | 119 (45.9) |  |
| Malay | 243 (29.7) | 113 (41.7) |  | 97 (37.5) |  |
| Indian | 159 (19.5) | 50 (18.5) |  | 43 (16.6) |  |
| Mother highest education groups |  |  | <0.001 |  | <0.001 |
| Below secondary | 44 (5.4) | 21 (7.7) |  | 22 (8.6) |  |
| GCE, ITE, Diploma | 530 (64.9) | 201 (74.2) |  | 182 (71.4) |  |
| University and above | 230 (28.2) | 44 (16.2) |  | 51 (20.0) |  |
| Parity |  |  | 0.094 |  | 0.906 |
| Nulliparous | 350 (42.8) | 105 (38.7) |  | 110 (42.5) |  |
| Multiparous | 467 (57.2) | 166 (61.3) |  | 149 (57.5) |  |
| Gestational weight gain (GWG) |  |  | 0.180 |  | 0.514 |
| Inadequate GWG by IOM | 84 (11.0) | 25 (10.3) |  | 29 (12.4) |  |
| Adequate GWG by IOM | 265 (34.7) | 74 (30.6) |  | 75 (32.2) |  |
| Excessive GWG by IOM | 414 (54.3) | 143 (59.1) |  | 129 (55.4) |  |
| Gestational diabetes mellitus (GDM) |  |  | 0.192 |  | 0.121 |
| GDM | 132 (16.2) | 38 (14.5) |  | 30 (12.7) |  |
| No GDM | 649 (79.4) | 224 (85.5) |  | 206 (87.3) |  |
| Maternal smoking groups |  |  | <0.001 |  | 0.037 |
| No exposure | 345 (42.2) | 100 (36.9) |  | 99 (41.8) |  |
| Exposed with cotinine level <level of detection | 216 (26.4) | 83 (30.6) |  | 80 (33.8) |  |
| Exposed with cotinine level <14 ng/ml | 112 (13.7) | 62 (22.9) |  | 45 (19.0) |  |
| Exposed with cotinine level >14 ng/ml | 30 (3.7) | 11 (4.1) |  | 13 (5.5) |  |
| Maternal age (years) | 30.3 (5.2) | 29.2 (5.5) | <0.001 | 29.3 (5.5) | <0.001 |
| Mother prepregnancy BMI category |  |  | 0.215 |  | 0.230 |
| BMI < 18.5 kg/m^2^ | 83 (11.2) | 31 (12.9) |  | 27 (11.7) |  |
| BMI 18.5-22.9 kg/m^2^ | 363 (48.9) | 104 (43.3) |  | 114 (49.6) |  |
| BMI 23.0-24.9 kg/m^2^ | 95 (12.8) | 33 (13.8) |  | 21 (9.1) |  |
| BMI ≥ 25.0 kg/m^2^ | 202 (27.2) | 72 (30.0) |  | 68 (29.6) |  |
| Fasting Plasma Glucose (mmol/L) | 4.4 (0.5) | 4.4 (0.6) | 0.023 | 4.4 (0.4) | 0.518 |
| 2 Hour OGTT Glucose (mmol/L) | 6.5 (1.5) | 6.3 (1.6) | 0.008 | 6.3 (1.4) | 0.013 |
| Offspring characteristics |  |  |  |  |  |
| Sex |  |  | 0.531 |  | 0.977 |
| Male | 439 (53.7) | 150 (55.4) |  | 139 (53.7) |  |
| Female | 377 (46.1) | 121 (44.6) |  | 120 (46.3) |  |
| Gestational age (weeks) | 38.8 (1.3) | 38.8 (1.2) | 0.680 | 38.9 (1.1) | 0.089 |
| Age on MRI day (days) | 9.7 (2.9) | 9.7 (2.9) | - | - |  |
| Birthweight (kg) | 3.1 (0.4) | 3.1 (0.4) | 0.516 | 3.1 (0.4) | 0.266 |
| Sum of skinfolds (mm) | 10.4 (2.3) | 10.6 (2.3) | 0.082 | 10.7 (2.0) | 0.017 |
| Fat mass by PEAPOD (kg) | 0.3 (0.1) | 0.3 (0.1) | 0.400 | 0.3 (0.1) | NA |
| Predicted fat mass (kg) | 0.3 (0.1) | 0.3 (0.1) | 0.519 | 0.3 (0.1) | 0.184 |
| Cord blood leptin (ng/mL) | 3.6 (3.1) | 3.6 (3.2) | 0.946 | 3.7 (3.0) | 0.368 |
| Cord blood adiponectin (µg/mL) | 7.1 (5.2) | 7.3 (6.9) | 0.070 | 7.3 (6.0) | 0.154 |

Data shown are N (%) for categorical variables or mean (SD) for continuous variables unless otherwise stated. Leptin and adiponectin concentrations are shown as median (interquartile range). Gestational weight gain (GWG) groups were defined by 1999 the Institute of Medicine (IOM) guideline for rates of weight gain in the second and third trimester per week. Prepregnancy BMI categories were defined by WHO recommendations for Asians. P values are based on between group comparison of study participants and non-participants using T-tests for continuous variables, Chi square tests for categorical variables and Mann-Whitney U tests for cord blood leptin and adiponectin concentrations.
